# Osimertinib plus anlotinib in untreated, EGFR-mutated, advanced non-small-cell lung cancer with concurrent alterations: a multicenter phase II study

**DOI:** 10.1093/oncolo/oyag344

**Published:** 2026-08-27

**Authors:** Xiaojun Yang, Qinquan Tan, Shiyuan Chen, Jieyun Xie, Yihong Zeng, Ruinian Zheng, Qinglin Tan, Wan Zhang, Churen Yu, Ziying Hong, Xinpei Yu, Guanming Jiang

**Affiliations:** Department of Thoracic Oncology, The Tenth Affiliated Hospital, Southern Medical University (Dongguan People’s Hospital), Dongguan, Guangdong, 523059, China; Department of Thoracic Oncology, The Tenth Affiliated Hospital, Southern Medical University (Dongguan People’s Hospital), Dongguan, Guangdong, 523059, China; Department of Thoracic Oncology, The Tenth Affiliated Hospital, Southern Medical University (Dongguan People’s Hospital), Dongguan, Guangdong, 523059, China; Department of Oncology, Guangzhou University of Chinese Medicine Dongguan Hospital (Dongguan Hospital of Traditional Chinese Medicine), Dongguan, Guangdong, 523059, China; Department of Thoracic Oncology, The Tenth Affiliated Hospital, Southern Medical University (Dongguan People’s Hospital), Dongguan, Guangdong, 523059, China; Department of Thoracic Oncology, The Tenth Affiliated Hospital, Southern Medical University (Dongguan People’s Hospital), Dongguan, Guangdong, 523059, China; Department of Thoracic Oncology, The Tenth Affiliated Hospital, Southern Medical University (Dongguan People’s Hospital), Dongguan, Guangdong, 523059, China; Department of Thoracic Oncology, The Tenth Affiliated Hospital, Southern Medical University (Dongguan People’s Hospital), Dongguan, Guangdong, 523059, China; Department of Oncology, Dongguan Houjie Hospital, Dongguan, Guangdong, 523960, China; Department of Pulmonary and Critical Care Medicine, The Affiliated Dongguan Songshan Lake Central Hospital, Guangdong Medical University, Dongguan, Guangdong, 523320, China; Department of Oncology, Guangzhou Institute of Cancer Research, The Affiliated Cancer Hospital, Guangzhou Medical University, Guangzhou, Guangdong, 510095, China; Department of Thoracic Oncology, The Tenth Affiliated Hospital, Southern Medical University (Dongguan People’s Hospital), Dongguan, Guangdong, 523059, China

**Keywords:** EGFR-mutation, concurrent, NSCLC, osimertinib, anlotinib

## Abstract

**Background:**

The standard first-line treatment for advanced EGFR-mutated non-small cell lung cancer (NSCLC) is EGFR tyrosine kinase inhibitors. However, concurrent mutations facilitate resistance evolution in EGFR-mutant lung adenocarcinoma and combination therapies may offer a superior approach. This study explores the efficacy and safety of osimertinib plus anlotinib as first-line treatment for advanced NSCLC with EGFR co-mutations.

**Materials and Methods:**

This study prospectively enrolled patients with advanced NSCLC harboring EGFR (19Del/21L858R) mutations and ≥1 mutation in TP53, PIK3CA, or RB1, who had not undergone systemic treatment. Kaplan-Meier survival analysis tested the differences among patients with different concurrent mutations. ctDNA test was administered before and after treatment and its correlation with efficacy.

**Results:**

A total of 38 patients were enrolled, with co-mutations in TP53 (76.3%), PIK3CA (26.3%), and RB1 (2.6%). With a median follow-up of 25.6 months, the median progression-free survival (PFS) was 29.0 months (95% CI, 18.6-NR), and 1-year PFS rate was 85% (95% CI, 70.4%-94.5%). Objective response rate was 73.7% (95% CI, 56.9%-86.3%), and disease control rate was 100.0% (95% CI, 90.7%-100.0%). Adverse events of any grade occurred in all patients. Seven (18.4%) patients experienced grade 3 treatment-related adverse events (TRAEs), with no grade 4 TRAEs or treatment-related deaths.

**Conclusion:**

Osimertinib plus anlotinib shows promising efficacy and manageable toxicity as first-line treatment for advanced NSCLC with EGFR co-mutations (ChiCTR2300070023).

**Clinical trial registration:**

This study was registered in the Chinese Clinical Trial Registry (ChiCTR) under the registration number ChiCTR2300070023 on March 31, 2023 (https://www.chictr.org.cn).

Implications for practice This study establishes a fully oral, chemotherapy-free regimen combining osimertinib and anlotinib as first-line therapy for EGFR-mutated lung cancer with concurrent mutations. Eliminating intravenous infusions enhances patient convenience while maintaining robust efficacy (median progression-free survival 29 months). These findings offer a less burdensome yet highly effective alternative, potentially transforming treatment experiences for this complex patient population.

## Introduction

Lung cancer remained the leading cause of cancer-related death worldwide in 2022, with an estimated 1.8 million fatalities.^1^ Non-small-cell lung cancer (NSCLC) accounts for approximately 85% of these cases, and adenocarcinoma is the most common histologic subtype. Although lung cancer represents only 12.4% of all newly diagnosed malignancies, it is responsible for 18.7% of cancer deaths, underscoring its aggressive biology and the inadequacy of current therapeutic strategies.

Epidermal growth factor receptor (EGFR)–activating mutations are the most frequent actionable drivers in NSCLC, with a prevalence of 40%-60% in East Asian patients.^2^ The widespread adoption of next-generation sequencing (NGS) has revealed that 20% of EGFR-mutant tumors harbor concurrent genomic alterations, most commonly TP53, KRAS, and PIK3CA.^3^ These co-mutations are associated with inferior prognosis and attenuated sensitivity to EGFR tyrosine-kinase inhibitors (TKIs). In the BENEFIT study, patients with EGFR and TP53 co-mutations achieved a median progression-free survival (mPFS) of only 4.6 months when treated with first-generation TKIs.^4^ A subsequent analysis confirmed that patients with co-mutations had significantly shorter PFS (mPFS: 5.7 vs 12.3 months, *P* = .02) and lower objective response rates (38% vs 89%, *P* < .001) than those with EGFR mutations alone.^5^ Even third-generation osimertinib, which yields remarkable efficacy in EGFR-mutant NSCLC, is markedly less effective in the presence of TP53, RB1, or KRAS co-alterations, owing to compensatory pathway activation and early adaptive resistance.^6^ Consequently, novel combination regimens are urgently needed for this molecularly defined, high-risk population.

The vascular endothelial growth factor (VEGF) pathway plays a pivotal role in tumor angiogenesis and TKI resistance.^7^ Binding of VEGF to VEGFR1/2 promotes endothelial proliferation and neovascularization, thereby remodeling the tumor microenvironment.^8^ Preclinical evidence indicates that EGFR blockade up-regulates VEGF expression, whereas combined inhibition of EGFR and VEGF signaling suppresses compensatory angiogenesis and restores TKI sensitivity.^9^ Experimental studies further demonstrate that the multi-target TKI anlotinib, when combined with osimertinib, re-sensitizes resistant cells in vitro and in vivo by suppressing AXL phosphorylation and modulating the c-MET/MYC/AXL axis.^10^

Clinically, the addition of anti-angiogenic agents to EGFR-TKIs has yielded consistent PFS benefits. In the open-label phase III NEJ026 trial (*n* = 228), mPFS was 16.9 months with erlotinib plus bevacizumab vs 13.3 months with erlotinib alone.^11^ CTONG1509, a nationwide, multicenter, randomized, open-label phase III study conducted in China (*n* = 311), demonstrated a mPFS of 18.0 months with bevacizumab plus erlotinib compared with 11.3 months with erlotinib monotherapy (hazard ratio, 0.55).^12^ Similarly, the global phase III RELAY study (*n* = 449) showed that ramucirumab plus erlotinib prolonged mPFS to 19.4 months vs 12.4 months with placebo plus erlotinib, with manageable toxicity.^13^ A preliminary study conducted by Yan involved 25 patients diagnosed with EGFR-mutated advanced or metastatic NSCLC who received a combination regimen of osimertinib and anlotinib as first-line treatment. The overall cohort’s PFS was 31.5 months (95% CI, 17.9-NE). Although PFS improvements have not uniformly translated into significant overall survival (OS) benefits, pre-specified subgroup analyses suggest a clear OS trend favoring the combination in patients with EGFR/TP53 co-mutation.^14^ These findings underscore the clinical value of combining EGFR-TKIs with anti-angiogenic therapy in concurrent NSCLC.

Anlotinib, an oral multi-target TKI approved in China for advanced NSCLC, inhibits VEGFR, PDGFR, and FGFR, thereby broadly suppressing tumor angiogenesis and proliferation.^15^ However, prospective evidence for osimertinib plus anlotinib in EGFR-mutant, co-mutation-positive NSCLC remains scarce.

We therefore initiated this single-arm, phase II study to evaluate the efficacy and safety of the all-oral combination of osimertinib and anlotinib in treatment-naive patients with EGFR-positive, concurrent advanced NSCLC. Serial NGS profiling of tumor tissue and circulating tumor DNA will be performed to identify predictive and prognostic biomarkers and to elucidate mechanisms of resistance.

## Materials and methods

### Study design and study population

This is a prospective, multicenter, single-arm phase II trial conducted in 3 hospitals in China. The main inclusion criteria are as follows: age between 18 and 75 years; ECOG performance status (PS) 0-2; expected lifetime at least 12 weeks; pathologically confirmed stage IV non-small cell lung cancer with measurable lesions according to RECIST 1.1 criteria, the long diameter of the tumor lesion on CT scan is ≥10 mm, and the short diameter of the lymph node lesion on CT scan is ≥15 mm (measurable lesions that have not received local treatments such as radiotherapy or cryotherapy); presence of EGFR mutations (19del or 21 L858R or T790M), and confirmed by NGS sequencing, also containing one or more of TP53, PIK3CA, RB1; no previous systemic treatment; patients who have previously received radiotherapy may be included, and previous radiotherapy must have ended at least 4 weeks prior to study enrollment, and any acute toxic reactions from previous radiotherapy must have resolved; locally irradiated lesions cannot be included in measurable lesions unless there has been significant progression of the lesion recorded after the last radiotherapy; normal function of major organs. The main exclusion criteria include: small cell lung cancer (including small cell carcinoma and mixed small cell and non-small cell lung cancer); presence of central tumors invading large vessels; or tumors showing obvious pulmonary cavitation or necrosis; or, based on imaging (CT or MRI), the researcher assesses that the tumor lesion is close to a large vessel with a risk of bleeding; only the presence of EGFR activating mutations (19del or 21 L858R or T790M) confirmed by NGS sequencing; patients with hypertension treated with two or more antihypertensive drugs in combination; abnormal coagulation function; significant clinical bleeding symptoms or a clear tendency to bleed within 3 months prior to inclusion; major surgery within 4 weeks prior to inclusion, etc. Complete list of inclusion and exclusion criteria. The study was conducted in accordance with the Declaration of Helsinki and Good Clinical Practice. The protocol and its amendments were approved by the independent ethics committees of each participating center. Written informed consent was obtained from each patient.

### Procedures

Eligible patients should take 80 mg of osimertinib orally once daily; anlotinib 10 mg, orally, once daily. Anlotinib is to be continuously administered for 2 weeks followed by a 1-week break, constituting a 3-week (21-day) cycle. Every two treatment cycles, efficacy is evaluated once. Patients who experience disease control and can tolerate adverse reactions should continue treatment. The study ends when the researcher deems the patient unsuitable for further treatment or assesses disease progression. Safety evaluations are conducted during the medication period. After discontinuation, survival follow-ups are performed every 2 months. All enrolled patients underwent baseline imaging, including contrast-enhanced CT and contrast-enhanced brain MRI. Imaging assessments were subsequently performed at least once every 12 weeks. All assessments were conducted by investigators in accordance with the Response Evaluation Criteria in Solid Tumors (RECIST), version 1.1. Adverse events are continuously monitored until 90 days after the last administration, and graded according to the National Cancer Institute’s Common Terminology Criteria for Adverse Events version 4.03. According to the protocol-defined dose modification criteria, a dose reduction of anlotinib to 8 mg/day was permitted. Treatment was permanently discontinued if the 8 mg/day dose was not tolerated. For the exploratory ctDNA analysis, blood samples were collected at baseline, at week 3 after treatment initiation, and every 12 weeks thereafter until disease progression, where feasible and following additional written informed consent. Next-generation sequencing was performed using the Oseq-ctDNA assay. Baseline tissue and plasma samples from a subset of patients were additionally analyzed using the Geneseq PRIME 437-gene panel.

### Outcomes

The primary endpoint of the study was the 12-month PFS rate, defined as the proportion of patients who did not experience disease progression or death from any cause within 12 months. Secondary endpoints included OS, PFS, investigator-assessed objective response rate (ORR), disease control rate (DCR) according to RECIST version 1.1, and safety indicators. OS is defined as the time from initial treatment to death from any cause; PFS is defined as the time from study entry to disease progression or death from any cause (for subjects lost to follow-up or at database lock who have not progressed, the last follow-up time is typically counted as the progression time); ORR is defined as the proportion of patients with CR or PR according to RECIST 1.1; DCR is defined as the proportion of patients with CR, PR, or SD according to RECIST 1.1. Adverse drug reactions are evaluated using the NCI CTCAE AE V4.0 criteria.

### Statistical analysis

With reference to the FLAURA study,^16^ the 12-month PFS rate for the overall population in the osimertinib group was 63.8%. Therefore, assuming that the osimertinib combined with anlotinib regimen increases the 12-month PFS rate to 85%, with *α* = 0.05 and *β* = 0.2, an enrollment period of 12 months, and a follow-up period of 12 months, the sample size was calculated using the exact binomial test method. Considering a dropout rate of 20%, this study plans to enroll 35 participants.

Statistical analysis was performed using R Statistical Software (version 4.1.3). Normally distributed continuous data were expressed as mean ± standard deviation, while non-normally distributed continuous data were presented as median (minimum, maximum). Categorical data were described using *n* (%), with 95% confidence intervals (CI) determined. The Kaplan-Meier method was used to illustrate PFS and OS, and safety analyses were primarily descriptive. Survival curve comparisons were performed using the log-rank test. A *P*-value < .05 was considered statistically significant.

## Results

### Patient characteristics

**Figure 1.**
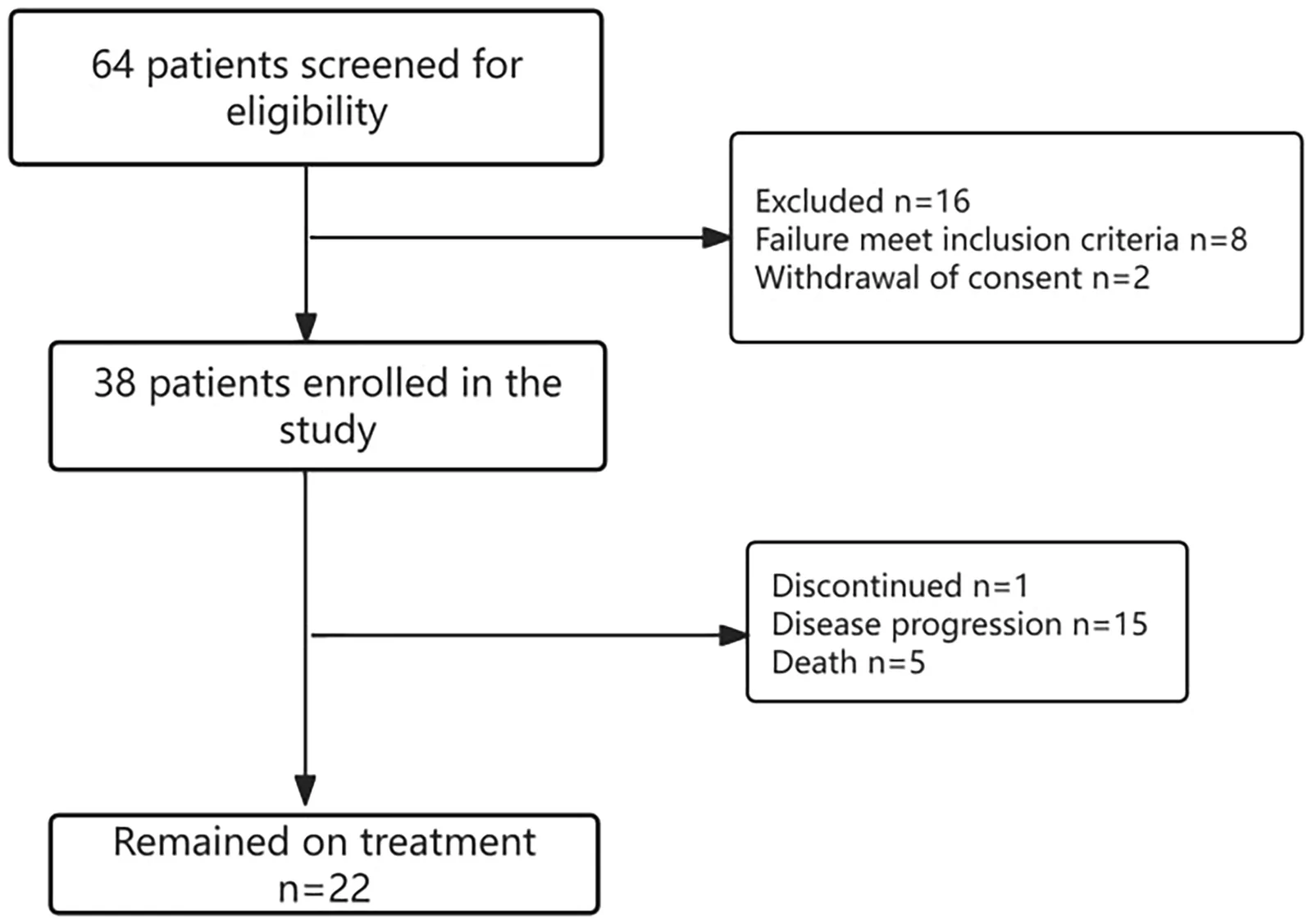
Patient flowchart.

From August 2021 to November 2023, 38 patients were enrolled in this study (Figure 1), and their characteristics were summarized in Table 1. The median age was 65.5 years (range: 42-76). The cohort comprised 39.5% males (*n* = 15) and 60.5% females (*n* = 23). Most patients were never-smokers (81.6%), while 15.7% were former smokers and 2.7% were current smokers. The majority had an ECOG Performance Status of 1 (78.9%), with 7.9% scoring 0 and 13.2% scoring 2. Clinically, 89.5% presented with stage IV disease, and 10.5% with stage IIIB/C. Nearly all patients (97.4%) had adenocarcinoma histology. EGFR mutations were evenly distributed between exon 19 deletions (47.4%) and L858R (52.6%). Co-mutations were frequent, including TP53 (76.3%), PIK3CA (26.3%), and rare RB1 (2.6%). Baseline metastatic sites included brain (47.4%), bone (44.7%), pulmonary (36.8%), pleural (34.2%), and liver (10.5%).

**Table 1.** Patient characteristics at baseline.

	Total (*n* = 38)
**Age-years, median (range)**	65.5 (42.0-76.0)
**Gender, *n* (%)**	
** Male**	15 (39.5%)
** Female**	23 (60.5%)
**Smoking status, *n* (%)**	
** Never**	31 (81.6%)
** Former**	6 (15.7%)
** Current**	2 (2.7%)
**ECOG PS, *n* (%)**	
** 0**	3 (7.9%)
** 1**	30 (78.9%)
** 2**	5 (13.2%)
**Clinical stage, *n* (%)**	
** IIIB/C**	4 (10.5%)
** IV**	34 (89.5%)
**Histology, *n* (%)**	
** Adenocarcinoma**	37 (97.4%)
** Others**	1 (2.6%)
**EGFR mutation, *n* (%)**	
** 19Del**	18 (47.4%)
** L858R**	20 (52.6%)
**Co-mutation, *n* (%)**	
** TP53**	29 (76.3%)
** RB1**	1 (2.6%)
** PI3KCA**	10 (26.3%)
**Metastases, *n* (%)**	
** Brain metastases**	18 (47.4%)
** Pulmonary metastases**	14 (36.8%)
** Pleural metastases**	13 (34.2%)
** Bone metastases**	17 (44.7%)
** Liver metastases**	4 (10.5%)

Abbreviation: ECOG PS: Eastern Cooperative Oncology Group Performance Status.

### Clinical response

**Figure 2.**
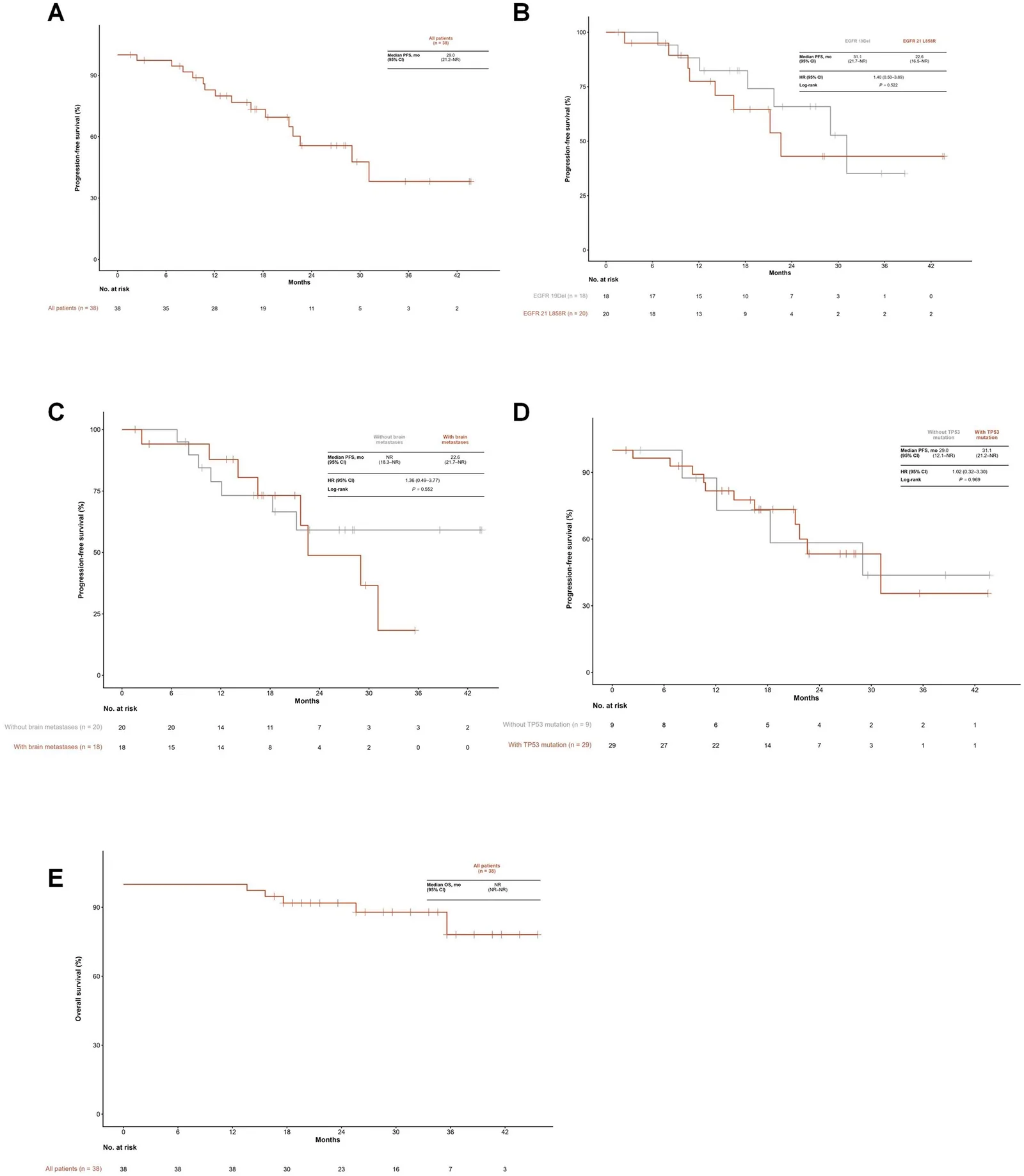
Kaplan–Meier curves of patients in different groups. (A) Progression-free survival of all study population. (B) PFS of patients harboring EGFR19Del or EGFR 21l858R. (C) PFS of patients with or without brain metastasis. (D) PFS of patients with TP53 mutation or without TP53 mutation. (E) Overall survival of all study population. PFS: progression-free survival; NR, not reached; CI, confidence interval; HR hazard ratio; OS: Overall survival.

With a median follow-up of 25.6 months (range: 15.6-43.6), 15 of 38 patients had disease progression or death; the mPFS was 29.0 months (95% CI, 21.2-NR, Figure 2A). PFS rate at 12 and 24 months were 85% (95% CI, 70.4%-94.5%) and 50.8% (95% CI, 35.3%-73.3%), respectively. Subgroup analyses, mPFS for EGFR subtypes 19DEL and 21L858R was 31.1 months (95% CI, 21.7-NR) and 22.6 months (95% CI, 16.5-NR) in Figure 2B, respectively (*P* = .52). According to baseline brain metastasis status, mPFS was 22.6 months (95% CI, 21.7-NR) in patients with brain metastases and was not reached in other patients (*P* = .55; Figure 2C). In subgroup analysis involving concomitant TP53 mutations, mPFS was 31.1 months (95% CI, 21.2-NR) and 29.0 months (95% CI, 12.1-NR) (*P* = .97) with or without mutation in Figure 2D. Regarding PIK3CA mutations, mPFS was 31.1 months (95% CI, 21.2-NR) compared to 22.6 months (95% CI, 18.3-NR) in patients without PIK3CA mutations, although the difference was not statistically significant (*P* = .43) in Figure S1. At the data cutoff, five deaths had occurred among 38 patients, corresponding to an OS data maturity of 13.2%. The Kaplan-Meier–estimated 24-month OS rate was 91.9% (95% CI, 83.4%-100%), and median OS was not reached (Figure 2E).

**Figure 3.**
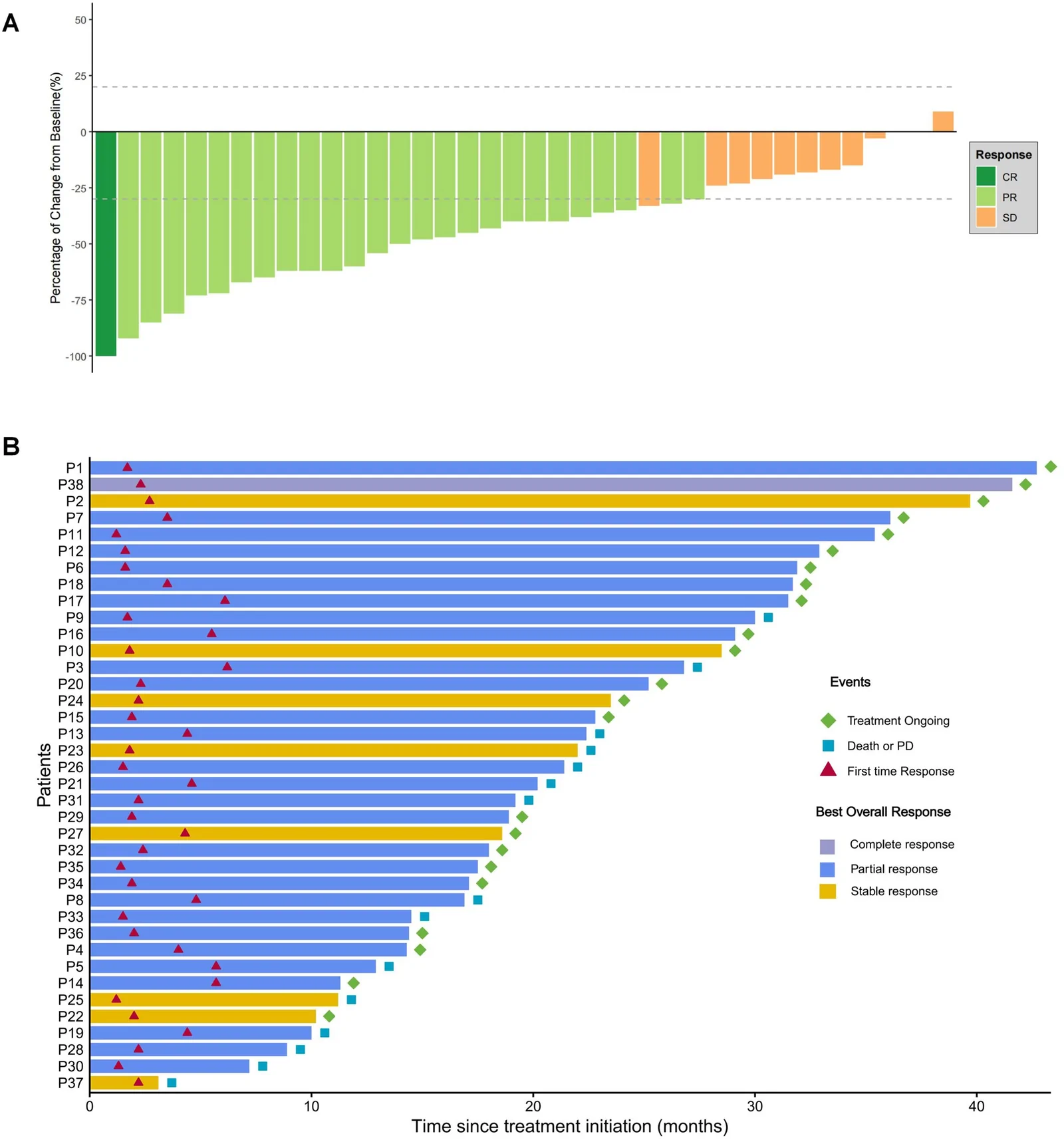
(A) Waterfall plots for responses per RECIST from baseline. Abbreviations: CR, complete response; PR, partial response; PD, progressive disease. (B) Swimmer plot of time on treatment.

ORR was 73.7% (95% CI, 56.9%-86.3%) and DCR was 100%(95% CI, 90.7%-100%; Table 2, Figure 3A). Among them, CR was achieved in 1 patient, PR in 27 patients, stable disease in 10 patients. The swimmer’s plot in Figure 3B illustrates the duration of treatment response in all patients. The waterfall plot in Figure 3 demonstrates the reduction in target lesion size from baseline, with a significant proportion of lesions showing marked shrinkage. Disease progression involved multiple sites: the primary tumor in 11 patients, brain metastases in 5, pleural effusion and pulmonary metastatic lesions in 3 each, and bone metastases in 2.

**Table 2.** Anti-tumor activity in patients.

	Total
**Best response, *n* (%)**	
** Complete response**	**1 (2.6%)**
** Partial response**	**27 (71.1%)**
** Stable disease**	**10(26.3%)**
**ORR, % (95% CI)**	**73.7%(95%CI,56.9%-86.3%)**
**DCR, % (95% CI)**	**100.0%(95%CI,90.7%-100%)**

Among the 18 patients with baseline brain metastases, 14 had measurable intracranial disease and were evaluable for intracranial response. The intracranial ORR was 85.7%, including five CR and seven PR, and the intracranial DCR was 92.9%, including one additional SD. All 18 patients were included in the intracranial PFS analysis, and five had experienced intracranial progression by the data cutoff. Median intracranial PFS was 22.8 months (95% CI, 21.3-NR; Figure S2).

Paired baseline and post-treatment plasma samples were available for 11 patients and were included in the exploratory ctDNA analysis. Eight patients achieved ctDNA clearance, whereas three had persistent detectable ctDNA. Median PFS was not reached in patients with ctDNA clearance and was 21.7 months in those without clearance (exploratory log-rank *P* = .017; Figure S3). Additional details are shown in Supplementary Results.

### Safety

Treatment-related adverse events (TRAEs) occurring in ≥10% of patients are summarized in Table 3. The most common any-grade TRAEs were rash (81.6%), hand-foot syndrome (HFS) (71.1%), hypertension (60.5%), abnormal liver function (47.4%), oral mucositis (50.0%), and decreased appetite (44.7%). Diarrhea occurred in 36.8% of patients. TRAEs were predominantly low-grade (Grade 1/2). The most frequent any-grade TRAEs (rash, hypertension, abnormal liver function) were exclusively Grade 1/2. Grade 3 or higher TRAEs were infrequent. HFS was the most common Grade ≥3 TRAE, occurring in 3 patients (7.9%). Single Grade 3 events (2.6% each) were reported for oral mucositis, diarrhea, decreased appetite, and nausea. Less frequent TRAEs (occurring in <10% of patients) included nausea (10.5% any grade), white blood cell decreased (10.5% any grade), anemia (7.9% any grade), bleeding or hemorrhage (5.3% any grade), platelet count decreased (5.3% any grade), hypercholesteremia (2.6% any grade), proteinuria (2.6% any grade), and embolism (2.6% any grade), all of which were Grade 1/2. No Grade 4 TRAEs or treatment-related deaths were observed. No cases of drug-related interstitial lung disease or toxic effects related to interstitial lung disease were reported. Adverse events leading to discontinuation of anlotinib were reported in 2 patients (5.2%). Adverse events leading to dose reduction of anlotinib were reported in 1 patient (2.6%).

**Table 3.** Summary of treatment-related adverse events.

TRAE	Any grade	Grade 1/2	≥Grade 3	Grade 4
**Hypertension**	23 (60.5%)	23 (60.5%)	0	0
**Rash**	31 (81.6%)	31 (81.6%)	0	0
**Hand-foot syndrome**	27 (71.1%)	24 (63.2%)	3 (7.9%)	0
**Oral mucositis**	19 (50.0%)	18 (47.4%)	1 (2.6%)	0
**Diarrhea**	14 (36.8%)	13 (34.2%)	1 (2.6%)	0
**Hypercholesteremia**	1 (2.6%)	1 (2.6%)	0	0
**Decreased appetite**	17 (44.7%)	16 (42.1%)	1 (2.6%)	0
**Abnormal liver function**	18 (47.4%)	18 (47.4%)	0	0
**Proteinuria**	1 (2.6%)	1 (2.6%)	0	0
**Nausea**	4 (10.5%)	3 (7.9%)	1 (2.6%)	0
**Bleeding or hemorrhage**	2 (5.3%)	2 (5.3%)	0	0
**Decreased white blood cell count**	4 (10.5%)	4 (10.5%)	0	0
**Anemia**	3 (7.9%)	3 (7.9%)	0	0
**Decreased platelet count**	2 (5.3%)	2 (5.3%)	0	0
**Embolism**	1 (2.6%)	1 (2.6%)	0	0

## Discussion

This study represents the first prospective investigation into the efficacy and safety of combining third-generation EGFR-TKI with an anti-angiogenic small molecule inhibitor in individuals harboring EGFR sensitizing mutations along with common co-mutations. The study successfully met its primary endpoint, achieving a 1-year PFS rate of 85%, with mPFS extended to 29.0 months. These findings highlight the significant potential of osimertinib in combination with anlotinib for treating EGFR-mutant populations with concomitant genetic alterations.

The evolving first-line treatment landscape for advanced EGFR-mutated NSCLC now includes combination strategies aimed at overcoming or delaying resistance. It has been demonstrated that the combination of EGFR and VEGFR pathways may lead to improved advances in tumor control in EGFR mutation-positive NSCLC vs targeting EGFR alone. In the 2021 ACTIVE Phase III study (*n* = 313), mPFS for apatinib plus gefitinib was 13.7 months vs 10.2 months for placebo plus gefitinib (HR = 0.71).^15^ Enrollment in the FL-ALTER Phase III trial of Zhou et al in 2024 had 315 patients and compared gefitinib plus anlotinib with placebo plus gefitinib. PFS was significantly prolonged (14.8 m vs 11.2 m, HR = 0.64, *P* = .003).^17^ The RAMOSE trial examines the activity and tolerability of osimertinib + ramucirumab vs osimertinib monotherapy in patients who were treatment naive for metastatic EGFR mutations (Ex19del or L858R) in NSCLC. In this randomized phase 2 study, among EGFR-mutated metastatic NSCLC patients who were initially treated with osimertinib, osimertinib plus ramucirumab significantly prolonged mPFS compared with osimertinib alone (24.8 vs 15.6 months, HR = 0.55, *P* = .023).^18^ It seems that compared with the combination with first generation EGFR-TKI, third-generation EGFR-TKI combined treatment strategy further improves survival benefit (3 months vs 9 months). New studies like FLAURA2 and MARIPOSA proved this. The FLAURA2 trial is a phase 3, international, open-label trial, examines osimertinib with or without chemotherapy in EGFR-mutated advanced NSCLC. Investigator-assessed progression-free survival was significantly longer in the osimertinib-chemotherapy group than in the osimertinib group (25.5 m vs 16.7 m, HR = 0.62, *P* < .001).^19^ Amivantamab plus lazertinib (amivantamab + lazertinib) has shown clinically meaningful and durable antitumor activity in patients with previously untreated or osimertinib-pretreated EGFR (epidermal growth factor receptor)-mutated advanced NSCLC. The median PFS was significantly longer in the amivantamab-lazertinib group than in the osimertinib group (23.7 vs 16.6 m, HR = 0.70, *P* < .001).^20^

While regimens such as osimertinib with chemotherapy or EGFR-MET bispecific antibody like amivantamab have demonstrated significant efficacy, the exploration of optimized, tolerable, and convenient combinations remains clinically relevant. In this context, our study demonstrates that the all-oral combination of osimertinib and anlotinib provides promising efficacy in patients with advanced EGFR-mutant NSCLC, achieving a median PFS of 29.0 months in the overall population, with particularly encouraging outcomes in key prognostic subgroups. Compared with FLAURA2 and MARIPOSA, the combination of osimertinib and anlotinib shows the longest mPFS. For patients with brain metastases at baseline, our study also shows promising efficacy. In our research, mPFS was 22.6 months with FLAURA2 PFS was 24.9 m, and MARIPOSA PFS was 18.3 m.

TP53 (55%-65%^21^)/PIK3CA (3%-8%, up to 25% in Asian populations^22^)/RB1 mutation (2%-3%^23^) is a high-frequency concurrent mutation with EGFR mutation and will shorten PFS and OS in some retrospective studies. In addition to the out-of-control of cell-cycle regulation and genomic instability caused by TP53 mutations,^24^ it also promotes the generation of tumor neovascularization. Forced expression of HIF-1α in tumor cells expressing p53 increases hypoxia-induced VEGF expression and enhances neovascularization and growth of tumor xenografts.^25^ The same RB1 mutation E2F-HIF-VEGF circuit and chromatin remodeling significantly pro-angiogenesis.^26^ PI3K/Akt/mTOR signaling pathway, the downstream pathway of EGFR, its activation can regulate cell proliferation, inhibit cell death, promote tumor angiogenesis and tumor invasion.^27^ The aforementioned genomic abnormalities are involved in multiple links of EGFR-TKI resistance. Anlotinib, a multi-target small molecule antiangiogenesis inhibitor, also inhibits multiple targets such as VEGFR/FGFR/PDGFR, showing better anti-tumor activity. EGFR-TKI combined with antiangiogenic drugs can delay or partially restore sensitivity by improving the tumor microenvironment and drug delivery, inhibiting proangiogenic factor-mediated drug resistance pathways. Moreover, anlotinib exhibits partial passive diffusion across cell membranes and inhibits activity of corresponding targets. In a study that established EGFR-mutated lung cancer cell lines expressing wild-type (WT) or various mutant p53 proteins using CRISPR-Cas9 technology, researchers found that TP53-GOF mutations promote early development of resistance to the EGFR-TKI osimertinib associated with sustained activation of ERK and expression of c-Myc.^28^ Osimertinib combined with anlotinib significantly inhibited growth of NSCLC-resistant tumors by co-mutant EGFR mutant TP53, and overcome drug resistance by promoting cytotoxic T cell infiltration and functional activation and reversing immunosuppressive microenvironment through VEGF-VEGFR blockade providing a potential biological mechanism for drug resistance in this type of population. All of the above studies show that EGFR-positive NSCLC with co-mutations is a potential population that could benefit from this regimen.

In this study, we enrolled EGFR-sensitive mutated NSCLC with a broader spectrum of co-mutations (TP53, PIK3CA, RB1). This study demonstrated that combination therapy of osimertinib and anlotinib resulted in a significantly prolonged mPFS of 29.0 months. In the FLAURA2 trial, among patients with TP53 co-mutations, osimertinib plus chemotherapy achieved a median PFS of 27.6 months. In the MARIPOSA trial, amivantamab plus lazertinib (ami + laz) showed a median PFS of 18.2 months in this subgroup. Notably, 47.4% of the enrolled patients had brain metastases at baseline, representing a clinically challenging population to treat. The PFS observed in the present study was comparable to that of the AUTOMAN trial,^29^ which enrolled a broader patient population and yielded a mPFS of 31.5 months, and appeared more favorable than that reported in the RAMOSE trial of osimertinib plus ramucirumab,^18^ in which the mPFS was 24.8 months. Combined with the aforementioned molecular characteristics related to co-mutation, we speculate that the potential synergistic mechanism between the two can be summarized into two aspects. First, anlotinib inhibits pathological neovascularization, and regulates the abnormally distorted tumor vascular network to a certain extent.^30^ This effect can optimize the tumor microenvironment, help osimertinib penetrate the blood-brain barrier and enter the tumor parenchyma more effectively, increase the drug concentration of the lesion, and reverse treatment failure caused by insufficient drug delivery. Second, bypass signaling activation, such as MET amplification or alternative angiogenic pathway activation, represents an important mechanism of acquired resistance to EGFR-TKIs.^31^ Through broad inhibition of multiple receptor tyrosine kinases, including VEGFR, anlotinib may suppress compensatory signaling pathways and delay the development of resistance. However, due to the limitations of cross-trial comparisons, including differences in patient characteristics and study designs, these results should be interpreted with caution. Even so, the findings of this study further support the potential activity of this combination regimen in high-risk populations with limited treatment options.

In an exploratory analysis of 11 patients with paired plasma samples, ctDNA clearance was associated with longer PFS than persistent ctDNA detection. This observation is consistent with the potential value of longitudinal ctDNA monitoring for assessing molecular response. However, the small sample size, limited number of progression events, and absence of a prespecified multivariable analysis preclude conclusions regarding the independent prognostic or predictive value of ctDNA clearance. These findings require validation in larger prospective cohorts.

Regarding safety, the toxicity profile of osimertinib plus anlotinib in our study was manageable. The most frequent TRAEs included rash (81.6%), hand-foot syndrome (71.1%), and hypertension (60.5%), which is consistent with the known profiles of each drug. Notably, only 18.3% of patients experienced ≥3 grade TRAEs, aligning with other studies of the intermittent dosing schedule of anlotinib (14 days on/7 days off), which adverse events were controllable. This profile may offer an advantage over regimens like amivantamab plus lazertinib, which is associated with higher rates of infusion-related reactions and dermatological toxicity, and osimertinib plus chemotherapy, which carries the burden of chemotherapy-related toxicities. Compared with 58.1% in the study by Wang et al.^32^, which utilized an osimertinib 80 mg plus anlotinib 12 mg regimen. In another study using anlotinib 8 mg combined with osimertinib, the incidence of grade ≥3 TRAEs was 10.7%.^33^ Furthermore, a phase Ib/II dose-escalation study conducted by Yan et al. reported that the proportions of treatment discontinuation due to anlotinib at different doses were 52%, 32%, and 24%, respectively. These findings suggest that an anlotinib dose of 10 mg may be a more appropriate choice in combination therapy to balance efficacy and safety.^14^ In this study, one patient withdrew from the study due to grade 3 cardiac dysfunction. The most common TRAEs associated with osimertinib plus anlotinib included fatigue, oral mucositis, diarrhea, and proteinuria, which may be related to anlotinib and are consistent with the previously established safety profile of anlotinib in real-world studies of NSCLC. Overall, the toxicity of this combination regimen was manageable and tolerable. Furthermore, the all-oral nature of the osimertinib-anlotinib regimen provides significant convenience, potentially improving patient quality of life and treatment adherence compared to intravenous combinations. This “chemo-free” strategy represents a valuable and convenient therapeutic option, particularly for patients seeking to avoid chemotherapy or intravenous infusions.

There are several limitations in this study. First, this is a single-arm study without a control group, leading to potential selection bias. Second, sample size is small, which limits the statistical efficacy of subgroup analyses (such as differences in efficacy of TP53/PIK3CA/RB1 comutant subgroups). Third, overall survival data may be immature and require longer follow-up to clarify the ultimate impact of combination therapy on survival. Fourth, the detection range of co-mutations may be limited due to sequencing depth and results.

This study was the first prospective exploration of the efficacy and safety of third-generation EGFR-TKI (osimertinib) combined with antivascular small molecule drug (anlotinib) in patients with advanced non-small cell lung cancer (NSCLC) carrying EGFR-sensitive mutations and high-frequency co-mutations (TP53/PIK3CA/RB1), and successfully reached the main endpoint. It provides a strong clue for advanced NSCLC patients with EGFR mutations combined with high-frequency co-mutation and brain metastasis, confirming that combined regimen has good efficacy and manageable safety in such refractory populations. In the future, the survival benefit of this program needs to be further verified through large-scale randomized controlled phase III trials, optimized screening of benefit populations (such as based on co-mutation spectrum or ctDNA clearance), and exploring its mechanism of overcoming drug resistance.

## Supplementary Material

oyag344_Supplementary_Data

## Data Availability

The data presented in this study are available upon request to the corresponding author.
