# Supplementary material for: Osimertinib plus anlotinib in untreated, EGFR-mutated, advanced non-small-cell lung cancer with concurrent alterations: a multicenter phase II study
Source: Oncologist. 2026 Aug 27;31(10):oyag344. doi: 10.1093/oncolo/oyag344 (PMC13585213; doi:10.1093/oncolo/oyag344)
Supplement: Supplementary file 1 [file oyag344_supplementary_data.docx]

**Supplementary Materials**

# Supplementary Results

**Dynamic analysis of peripheral blood ctDNA**

We analyzed the genomic data based on blood samples for circulating tumor DNA (ctDNA) analysis which were available for 11 patients at baseline,11 patients monitored via next-generation sequencing (NGS), ctDNA clearance post-treatment was associated with superior PFS (mPFS: NR vs. 21.7 months, P=0.02, **Figure S3**). The molecular dynamics of EGFR-sensitive mutations (L858R and 19del) in ctDNA samples were tracked throughout the treatment course. These mutations exhibited a decrease or were undetectable at Cycle 3, followed by an increase or re-emergence upon disease progression. Notably, 54.5% (6/11) of patients achieved clearance of EGFR-sensitive mutations, and the mPFS for patients with and without clearance of EGFR mutations had not been reached.

# Supplementary Figures

**Figure S1. Kaplan–Meier curves of patients in patients with PI3KCA mutation or without PI3KCA mutation.** PFS, progression-free survival; NR, not reached; CI, confidence interval; HR hazard ratio.

**Figure S2. Kaplan**–**Meier intracranial progression-free survival (iPFS) curve for evaluable patients with brain metastases.** iPFS: intracranial progression-free survival

**Figure S3. Molecular analysis of peripheral blood ctDNA.**

(A) Landscape of concurrent mutations in the intention-to-treat population. CR, complete response; PR, partial response; PD, progressive disease. (B) The Kaplan–Meier curves of PFS of patients with or without ctDNA clearance.
